# Sleep and inflammatory markers in different psychiatric disorders

**DOI:** 10.1007/s00702-015-1492-3

**Published:** 2015-12-09

**Authors:** Krzysztof Krysta, Marek Krzystanek, Agnieszka Bratek, Irena Krupka-Matuszczyk

**Affiliations:** 1 Department of Psychiatric Rehabilitation, Medical University of Silesia, ul. Ziołowa 45/47, 40-635 Katowice, Poland; 2 Department of Psychiatry and Psychotherapy, Medical University of Silesia, ul. Ziołowa 45/47, 40-635 Katowice, Poland

**Keywords:** Sleep, Inflammatory markers, Psychiatric disorders

## Abstract

Many psychiatric disorders, like schizophrenia, affective disorders, addictions and different forms of dementia are associated with sleep disturbances. In the etiology and course of those diseases inflammatory processes are regarded to be an increasingly important factor. They are also a frequently discussed element of the pathology of sleep. In this literature review reports on correlations between poor sleep and inflammatory responses in various psychiatric conditions are discussed. The link between schizophrenia, affective disorders and inflammatory cytokines is a complex phenomenon, which has been already confirmed in a number of studies. However, the presence of sleep deficits in those conditions, being a common symptom of depression and psychoses, can be an additional factor having a considerable impact on the immunological processes in mental illnesses. In the analyzed data, a number of studies are presented describing the role of inflammatory markers in sleep disturbances and psychopathological symptoms of affective, psychotic, neurogenerative and other disorders. Also attention is drawn to possible implications for their treatment. Efforts to use, e.g., anti-inflammatory agents in psychiatry in the context of their impact on sleep are reported. The aspect of inflammatory markers in the role of sleep deprivation as the treatment method in major depressive disorder is also discussed. A general conclusion is drawn that the improvement of sleep quality plays a crucial role in the care for psychiatric patients.

## Introduction

The correlation between sleep quality and immunological processes has been confirmed in a number of research reports (Kapsimalis et al. 2005; Krueger 2008; Krueger et al. 2011; Weschenfelder et al. 2012; Zeitzer 2013). The changes in the activity of inflammatory factors in related sleep disorders may be linked to the functioning of the central nervous system (Wisor et al. 2011; Zhu et al. 2012; Kang et al. 2013; Zielinski et al. 2014). Appropriate length and quality of sleep is a commonly discussed lifestyle factor affecting the functioning of healthy subjects, and the role it plays in the course of various psychopathological conditions is meaningful (Prather et al. 2009). The correlation between sleep disturbances and immunological changes, which may affect the brain functioning seems to have a key impact on the development and course of different psychiatric disorders (Illi et al. 2012; Voderholzer et al. 2012) as well as of comorbid conditions, which may occur (Wichniak and Jarema 2012). The link between depression or schizophrenia and inflammatory cytokines is a complex phenomenon, which has been already confirmed in a number of studies (Khandaker et al. 2014; Maes et al. 1995; Potvin et al. 2008; Drexhage et al. 2010; Miller et al. 2011), which provide evidence indicating that increased levels of pro-inflammatory cytokines play an important role in the etiology of schizophrenia and depression. However, the presence of sleep disturbances in those conditions, being a common symptom of the syndromes of depression and psychoses, can be an additional factor having a considerable impact on the immunological processes in psychiatric disorders.

The aim of this literature analysis is to find out the correlation between sleep disturbances, inflammatory processes and mental conditions like affective disorders, schizophrenia, addictions, dementia and other psychiatric disorders.

## Methods

The focus of this review was on studies on sleep, psychiatric disorders and inflammatory processes (1970–2014). We searched PubMed using the following search terms(effective date: 1st August 2015): (sleep (Title/Abstract) AND psychiatric disorders (Title/Abstract) OR depression (Title/Abstract) OR schizophrenia (Title/Abstract) OR addiction (Title/Abstract) OR dementia AND inflammatory processes (Title/Abstract) OR cytokine (Title/Abstract) OR interleukin OR tnf OR prostaglandins OR immune system AND English (lang) AND: 2015/08/01(PDAT)).

## Results

We retrieved 38 results from our PubMed search. We drew our attention mainly to those of them, which were original study papers devoted to groups of patients suffering from schizophrenia, depression, addictions, dementia, reaction to severe stress, adjustment disorders and psychosomatic disorders complicated by serious sleep disturbances, and in which the analysis of inflammatory markers was taken into consideration. Finally we found ten papers matching our criteria, which are listed and summarized in Table 1. Those papers differed in terms of study designs and methodology, with the inclusion of various clinical study groups with the number of participants within a range from 14 to 95. The most common markers evaluated in those studies were IL-1β, IL-6, IL-10, Prostaglandin D2, E2, F2α, corticotropin, IFN-gamma, NK cells, and TNF.

**Table 1:** Studies on inflammatory markers in schizophrenia, depression, addictions, dementia, reaction to severe stress, adjustment disorders and psychosomatic disorders complicated sleep disturbances

Authors (year)	Study group	Inflammatory factor	Conclusions
Prather et al. (2009)	95 non-depressed hepatitis C patients	IFN-alpha, IL-6	High levels of inflammation and poor sleep quality may be risk factors for IFN-alpha induced depression
Motivala et al. (2005)	22 patients with depressive disorder	IL-6, sICAM, MCP-1, IL-6sR	Nocturnal elevations of IL-6 and sICAM associated with sleep disturbance in depressed patients
Appelberg et al. (1997)	20 drug-free patients with acute non-affective psychoses	IL-1β in plasma	Negative correlation between IL-1β and the length of sleep period and of REM sleep Positive correlation between IL-1β levels and REM latency
Nishino et al.(1998)	14 unmedicated schizophrenic patients	Prostaglandin D2, E2, F2α and corticotropin releasing factor in cerebrospinal fluid	No correlations between inflammatory factors and sleep parameters
Heffner et al. (2012)	45 women and 38 men aged 50 years and older undergoing acute stress	IL-6	Poor sleepers had significantly larger IL-6 responses to the cognitive stressors than good sleepers
Von Känel et al. (2006)	64 older caregivers of people with AD	IL-6, D-dimer	Poor sleep was associated with higher plasma IL-6 and D-dimer levels
Chen et al. (2012)	43 drug-free AD patients	IL-1β and TNF-α	Daytime sleepiness in mild and moderate AD patients is associated with elevation of serum TNF-α
Redwine et al. (2003)	24 alcoholic patients	IFN-gamma, IL-10, IL-6, NK cells	Disordered sleep contributes to immune alterations in patients with chronic alcoholism
Irwin et al. (2004)	16 abstinent African American alcoholics	IL-6 and TNF	Circulating levels of proinflammatory cytokines may have a negative influence on sleep initiation
Heffner et al. (2011)	25 adults with chronic low back pain	IL-6	In adults with chronic low back pain poorer sleep quality was associated with higher IL-6 levels

Some other papers were either reviews, like the works of Ritter et al. (2013), Motivala et al. (2011) or referred to wider phenomena concerning inflammation processes and psychiatric disorders, anyway they also supported our discussion.

## Discussion

### Sleep, depression and cytokines

Brain, belonging to cytokine regulatory pathways, regulates the leukocyte action via autonomic nerves and neuroendocrine hormones (Besedovsky et al., 1983). Some cytokines like IL-1 are produced by astrocytes and microglia cells (Merill 1992) and the increased IL-1 level in the blood of depressive patients has been confirmed. IL-1 in CNS is known to be responsible for psychomotor retardation. Moreover, there are findings, suggesting that increased secretion of IL-6 is involved in the pathogenesis of depression. The increased level of IL-6 has been shown in patients with depressive disorder (Maes et al. 1995) and it probably influences the metabolism of serotonin in CNS, decreasing the availability of tryptophan in the blood (Fernstrom and Faller 1978). Many authors conclude that cytokine pathways are responsible for mutual influence of poor sleep and inflammatory cytokines (Meier-Ewert et al. 2004, Irwin et al. 2006). Accumulative evidence suggests cross relations between sleep, depression and disturbances of inflammatory system.

Recently, two main streams of studies are distinguished. One focuses on the relation between mechanisms of sleep and inflammatory processes by examining the influence of induced sleep disturbances on inflammatory markers. It is proven that total sleep deprivation induces the changes in inflammatory markers, which suggests the relation between sleep and inflammatory cytokines. Two findings are observed: the increase of CRP in total sleep deprivation, and the increase of IL-6 in longer periods of partial sleep deprivation (Meier-Ewert et al. 2004; Vgontzas et al. 2004). This pattern of changes in inflammatory markers occurs only if the sleep deprivation lasts longer than one night. In shorter human experiments the results were not so consistent (reviewed in Motivala 2011). However, even 1 day of partial sleep deprivation produces many short lasting disturbances in immunity, like reduction of activity of natural killers and lymphokine-activated killers or the suppression of IL-2 production (Irwin et al. 2006). The important implication from human studies is that adequate sleep period shortens the time of viral infection (Cohen et al. 2009). Due to the measurement difficulties, studies of another inflammatory cytokines like TNFa and IL-1b are inconsistent (reviewed in Motivala 2011). New studies reveal the relation between circadian rhythm and immunological system activity (Porkka-Heiskanen et al. 2013). Going further, loss of sleep triggers the genetic mechanism of transcription of genes related to immunity (Moller-Levet et al. 2013).

The other approach identifies the connection between sleep and inflammatory processes in observation of how inflammatory cytokines behave in insomnia. It was reviewed by Motivala (2011) that chronic sleep disturbances lead to elevation of inflammatory cytokines like IL-6, CRP and TNFa. The authors indicate no clear evidence that insomnia is the only reason of inflammatory cytokines disturbances and the importance of more thorough analysis of comorbidity in the future studies.

Because of the high prevalence of poor sleep in depressive patients (90 %) the sleep disturbances may link depression with the increase of inflammatory cytokines. Prather et al. (2009) gave interferon alpha (IFNα) to the non-depressive patients with hepatitis C. He observed major depression in 22 % of 95 patients as well as the significant decrease of the sleep quality (Prather et al. 2009). Statistical analysis revealed the time sequence of the observed phenomena. The higher pre-treated blood concentration of IL-6 was the risk factor of the development of major depressive disorder but sleep disturbances was followed by depressive symptoms. One of the most important conclusions from the study is that inflammatory markers disrupt sleep and only then disturbed sleep results in depressive symptoms (Buysse et al. 2008). The conclusion is consistent with the previous findings of Motivala et al. (2005) who showed that sleep disturbances evoke the increase of IL-6 and soluble intercellular adhesion molecule, and that the depression relates to the problems with the initiation of sleep (Motivala et al. 2005). The described phenomena are present not only in major depression, but also in other affective disorders. According to Ritter et al. (2013) changes observed in the sleep of patients with bipolar disorder are also related to the elevation of IL-6.

Most recent extensive meta-analysis of Howren and colleagues (2009) aimed at finding the positive relation between depression and inflammatory markers in scientific papers published from January 1967 to January 2008 (Howren et al. 2009). The authors concluded that there is a positive correlation between depression and the increase of the three inflammatory cytokines: CRP, IL-1 and IL-6. More importantly, the revealed associations are not related to medication, age or gender of the patient. Another research, done by a Canadian group found changes in levels of serotonin, pro-inflammatory cytokines, brain-derived neurotrophic factor (BDNF) and other transmitters in comorbid chronic pain, depression and sleep disruption (Boakye et al. 2015). The presence of a correlation between cytokines level and depressive symptoms may justify the proposal of the new type of depression called inflammatory cytokine-associated depression (ICAD) (Lotrich 2015).

Another aberration of immunity in depressive patients is aforementioned disturbed natural killers’ (NK) activity. In depressed patients a bigger severity of insomnia correlates with lower NK activity (Irwin 2002). More importantly, the disturbed NK function does not relate to any other depressive symptom. Similar pattern of disturbance was found in non-depressed insomnia suffering subjects, which implies strong relation between immunity and sleep but not with depression itself (Irwin 2002).

### Inflammatory factors and sleep disturbances in schizophrenia

Sleep disturbances, although seldom reported as a predominant complaint, are one of the hallmarks of schizophrenia. Disturbed sleep is estimated to occur in 30–80 % of people with schizophrenia, depending on the degree of psychotic symptomatology (Cohrs 2008). The main polysomnographic features in schizophrenia are: reduced sleep efficiency; decreased total sleep time; shortened slow wave sleep, REMOL (rapid eye movement sleep onset latency) and REM (rapid eye movement) sleep; increased sleep onset latency and longer wake time after sleep onset (Monti et al. 2013). According to a case–control study by Wullf and colleagues (2012), delayed sleep onset latency, disturbance of sleep maintenance and prolonged time awake are characteristics for schizophrenic patients regardless of either their medication status (drug-naive or drug-treated) or the phase of the clinical course (acutely psychotic or clinically stable). Sleep disturbances observed in schizophrenic patients could be partially related to the hyperactivity of the dopaminergic system (Monti et al. 2013). Although there is a general consensus that the dopaminergic neurotransmission is involved in the pathophysiology of schizophrenia, the role of the inflammatory process in the pathogenesis of schizophrenia has been discussed since almost a century (Dameshek 1930). A growing body of evidence indicates increased levels of pro-inflammatory cytokines in schizophrenia patients (Potvin et al. 2008; Drexhage et al. 2010; Miller et al. 2011). Among them, IL-6, IL-10 and TNFα (Cazullo et al. 1998; Kunz et al. 2011; Miller et al. 2011) gained special research attention. It was observed that the serum levels of IL-2 and IL-6 are increased in first episode drug-naïve patients with psychosis (Petrikis et al. 2015), and that IL-3 levels are significantly increased in patients with chronic schizophrenia (Xiu et al. 2015). Brazilian authors go far enough in their analyses to postulate that the combination of five biomarkers (sTNF-R1, sTNF-R2, CCL11, IP-10, IL-4) may predict the diagnosis of SCZ with a sensitivity of 70.0 % and a specificity of 89.4 % (Noto et al. 2015). According to the Australian group there can be a subtype of schizophrenia including patients displaying poor verbal fluency and reduced Broca’s area volume, in whom the IL-1β mRNA cytokine level is elevated (Fillman et al. 2015). On a different note, maternal prenatal infections were found to increase the risk of schizophrenia, regardless of the pathogen (Brown and Derkits 2010). Neuroinflammation during early fetal development caused by exposure to cytokine-releasing agents may be pathophysiologically relevant to the progression of schizophrenia (Meyer et al. 2011). Additionally, associations were found between schizophrenia and inflammation-related gene regions. In a genome-wide association study (Shi et al. 2009) most probable susceptibility genes were identified in a region on chromosome 6p22.1, which includes a histone gene cluster and several immunity-related genes. Hence, the question emerges whether any evident link could be found between inflammatory factors and sleep disturbances in schizophrenia. Appelberg et al. (1997) performed polysomnography and measured morning IL-1β plasma values in 20 drug-free patients with acute non-affective psychoses (10 diagnosed with schizophrenia, 5 with delusional disorder and 5 with atypical psychosis). The authors found that the length of sleep period and the relative time of REM sleep correlated negatively with IL-1β levels. There was also a positive correlation between REM latency and IL-1β levels. Another study (Nishino et al. 1998) was aimed to shed a light on the role of corticotropin releasing factor (CRF) and prostaglandins (PGs) in the pathophysiology of sleep disturbances observed in schizophrenia. The study hypothesis was based on findings of previous studies, in which both CRF (Ehlers et al. 1986; Opp et al. 1989) and PGs (Hayaishi 1991; Matsumura et al. 1994) were reported to modulate sleep in experimental animals. To test that hypothesis, the authors carried out polysomnographic recordings and measured the cerebrospinal fluid levels of PGD2, PGE2, PGF2α and CRF in 14 unmedicated schizophrenic patients and 14 healthy controls. However, the results were not in favor of authors’ assumptions—neither group differences in cerebrospinal CRF and PG levels, nor correlations between CSF variables and sleep parameters were reported. Melatonin comprises an indirect link between sleep disturbances and inflammation in schizophrenia. Hypnotic and circadian rhythm resynchronizing features of melatonin are widely acknowledged. Nevertheless, it is noteworthy that melatonin is additionally involved in immunomodulation (Maldonado et al. 2009), attenuates pro-inflammatory cytokines and other inflammatory mediators, and is acting as a free radical scavenger (Esposito and Cuzzocrea 2010). Melatonin was found to be an important factor in the etiology, pathogenesis and treatment of schizophrenia (for review, see Anderson and Maes 2012). Accumulating evidence indicates reduced levels in patients with schizophrenia, regardless of the treatment status (Monteleone et al. 1992, 1997; Bersani et al. 2003). A case–control study conducted among monozygotic twins discordant for schizophrenia reported significant group differences in levels of melatonin (Afonso et al. 2010). The question whether melatonin alterations are closely related to immunological changes observed in schizophrenic subjects still remains open.

### Inflammatory factors and sleep disturbances in other psychiatric disorders

The literature referring to the problem of inflammatory disturbances in sleep disorders other than depression and schizophrenia is not very rich; however, some interesting observations are available. Inflammatory responses to sleep disorders can be found for example in patients undergoing acute stress, even generally healthy. An interesting study relating to this group was performed by Heffner et al. (2012) on men and women aged 50 and older. The patients with sleep disorders were examined with a series of complex cognitive tests, and it turned out that they had significantly larger IL-6 responses to the cognitive stressors in comparison with participants with no reported sleep problems. The authors raised a conclusion that older adults may present vulnerability specific to changes in sleep and inflammatory regulation related to age. A similar phenomenon can be observed among caregivers of patients suffering from dementia. Von Känel et al. (2006) attempted to determine the correlation between sleep measures and plasma levels of cytokine interleukin (IL-6) and the procoagulant marker fibrin d-dimer in those group of people. The results showed that this correlation was positive, especially in caregivers of patients with Alzheimer’s disease. The above problem was raised also by Mills et al. (2009). Their study examined the effects of caregiver gender and severity of the spouse’s dementia on sleep, coagulation, and inflammation in the caregiver. The results of their study suggested that males, who take care of spouses with more severe dementia experience more disturbed sleep and have increased coagulation processes. This phenomenon was associated with disturbed sleep. Inflammatory responses to sleep deficits can also be present in the patients suffering from dementia themselves. Chen et al. (2012) performed a study aimed to examine whether sleep disturbance in a cohort of patients with mild/moderate Alzheimer’s disease was associated with serum levels of IL-1β and TNF-α. The results indicated that that daytime sleepiness in mild and moderate AD patients is associated with elevation of serum TNF-α concentrations.

Another interesting group of patients among whom sleep disorders may lead to changes in inflammatory factors are patients with addiction. Redwine et al. (2003) examined the relationships between sleep, nocturnal expression of immunoregulatory cytokines, and natural killer (NK) cell activity in alcoholic patients as compared with control subjects. Alcoholic patients showed lower levels of IL-6 production, suppression of the IL-6/IL-10 ratio, and a reduction of NK cell activity, coupled with losses of delta sleep and increases of rapid eye movement sleep, as compared with control subjects, which led to a conclusion that disordered sleep contributes to immune alterations in patients with chronic alcoholism. Irwin et al. (2004) examined abstinent African American alcoholics. Prolonged sleep latency and increased rapid eye movement sleep in those patients was correlated with the elevations of IL-6 and TNF as compared to controls after adjustment for alcohol consumption and body mass index. As a result of sleep deprivation, patients addicted to alcohol showed greater nocturnal levels of IL-6 and greater nocturnal increases of TNF in comparison with control group members. The authors concluded that that circulating levels of proinflammatory cytokines may have a negative influence on sleep initiation. Changes in inflammatory factors can be found also among patients with psychosomatic disorders. Ali and Orr (2014) report that there is an association of sleep disturbances with the disease activity, subclinical inflammation, and risk of disease relapse in inflammatory bowel disease. Heffner et al. (2011) performed a study, the objective of which was to examine associations between sleep disturbance and interleukin-6 (IL-6), in patients with and without chronic low back pain. It turned out that they had more sleep disturbance than the members of the control group, suggesting that inflammatory processes may play a significant role in the cycles of pain and sleep disturbance. The authors expressed their hope that clinical interventions improving sleep and reducing coexisting inflammatory dysregulation could improve chronic pain management.

### Conclusions and implications for new treatment options

The above analysis of literature data shows that a link can be found between psychiatric disorders, sleep and inflammatory processes. This link is important for further research on the etiology of mental illnesses, but may also turn out to be useful in new future treatment strategies, e.g., anti-inflammatory agents may possibly be useful both in sleep disorders, and in mental illnesses. An interesting study was performed on mice undergoing sleep deprivation, treated with minocycline. This treatment strategy showed to have an impact on sleep architecture; however, the authors did not discover the underlying mechanism (Wisor et al. 2011). Attempts are made to use minocycline in affective disorders. Miyaoka et al. (2012) in their 6-week, open-label study found out that minocycline in combination with antidepressants is effective and well tolerated in the treatment of unipolar psychotic depression. Chaudhry et al. (2012) observed a positive effect of minocycline on symptoms of schizophrenia. Also other research projects were carried out in this field (Dean et al. 2012; Savitz et al. 2012; Berk et al. 2013; Jhamnani et al. 2013; Fond et al. 2014). The above results suggest that anti-inflammatory agents could be useful in the treatment of both sleep disorders, and such psychiatric conditions like depression or schizophrenia, being useful also in situations, when these conditions are combined. In this context it is also important to evaluate how sleep deprivation, which is used as one of treatment options in major depressive disorder, affects inflammatory processes. This problem was widely discussed in the paper by Voderholzer et al. (2012). According to the authors sleep deprivation affect cytokine levels in both depressed patients and healthy subjects, but does it in different ways. In depressed patients IL-6 levels are normalized during the recovery night, after earlier increase. The treatment proposals, which were mentioned above may turn out to be advantageous for patients suffering from psychiatric disorders with additional sleep disturbances; however, still more researches are necessary to conform their efficacy. Anyway it should be important for clinicians, that the improvement of sleep quality may be a key factor in the process of treatment of these diseases.
